# Short-term passenger flow prediction for urban rail systems: A deep learning approach utilizing multi-source big data

**DOI:** 10.1371/journal.pone.0333094

**Published:** 2025-10-06

**Authors:** Hongmeng Cui, Bingfeng Si, Dazhuang Chi, Yueqing Li, Ge Li, Yuanmeng Chen

**Affiliations:** 1 School of Systems Science, Beijing Jiaotong University, Beijing, China; 2 School of Traffic and Transportation, Beijing Jiaotong University, Beijing, China; 3 School of Traffic and Transportation Engineering, Dalian Jiaotong University, Dalian, Liaoning, China; 4 School of Traffic and Transportation, Xi’an Railway Vocational and Technical Institute, Xi’an, Shaanxi, China; National Institute of Technology Rourkela, INDIA

## Abstract

Predicting short-term passenger flow in urban rail transit is crucial for intelligent and real-time management of urban rail systems. This study utilizes deep learning techniques and multi-source big data to develop an enhanced spatial-temporal long short-term memory (ST-LSTM) model for forecasting subway passenger flow. The model includes three key components: (1) a temporal correlation learning module that captures travel patterns across stations, aiding in the selection of effective training data; (2) a spatial correlation learning module that extracts spatial correlations between stations using geographic information and passenger flow variations, providing an interpretable method for quantifying these correlations; and (3) a fusion module that integrates historical spatial-temporal features with real-time data to accurately predict passenger flow. Additionally, we discuss the model’s interpretability. The ST-LSTM model is evaluated with two large-scale real-world subway datasets from Nanjing and Chongqing. Experimental results show that the ST-LSTM model effectively captures spatial-temporal correlations and significantly outperforms other benchmark methods.

## Introduction

With the rapid development of urban rail transit (URT) systems, there has been increasing focus on their intelligent construction in both practice and academia. Short-term metro passenger flow prediction, a critical task within intelligent transportation systems (ITS), has garnered significant research attention due to its practical impact on operators and passengers alike. For metro operators, reliable short-term passenger flow predictions support daily network management and optimize train schedules. Additionally, accurate predictions aid in appropriate station staffing and can guide passenger evacuation in advance, mitigating or preventing accidents that might result in significant casualties. Passengers, on the other hand, can use these predictions to make informed travel decisions, effectively planning their routes and travel times to enhance their travel experience.

Over the years, various models for short-term passenger flow prediction have been proposed, ranging from classical statistical methods to advanced artificial intelligence approaches. The rapid development of big data technology has recently expanded the sources of urban traffic data, offering new opportunities for detailed studies of passenger travel patterns. With access to extensive data and enhanced computing power, deep learning methods have made significant strides [1,2]. Despite the growing adoption of deep learning in the transportation domain [3,4], effectively applying these techniques to URT passenger flow prediction remains challenging due to the system’s inherent complexity.

In this study, based on a systematic analysis of existing approaches, we identify three key challenges in applying deep learning to short-term passenger flow prediction in URT systems: how to mitigate the influence of weakly correlated historical data, how to effectively model spatial dependencies, and how to determine appropriate prediction intervals.

Short-term passenger flow prediction can be viewed as a time-varying time series prediction problem, emphasizing the importance of analyzing temporal correlations among variables. Temporal correlation pertains to the evolving relationships of passenger flow across different time intervals and the interactions and influences between these intervals. Current methods typically utilize continuous historical data for predictions [5,6]. However, this approach often integrates weakly correlated information from days with different travel patterns. For example, significant disparities exist in passenger travel behaviors between weekdays and weekends. Failure to distinguish these distinctions may introduce noise into the prediction process, potentially compromising accuracy.

Meanwhile, the Long Short-Term Memory (LSTM) network—a widely used deep learning model—has demonstrated strong capability in modeling temporal dependencies from sequence data [7]. However, a significant drawback of LSTM is its focus on temporal data correlations, often neglecting spatial correlations—the inherent connections and mutual influences between passenger flows at different stations. This limitation can significantly impact prediction accuracy. Therefore, exploring methods for effectively capturing spatial correlations within URT systems is crucial.

Moreover, prior studies have used various time intervals ranging from 5 minutes to 1 week for prediction. Yet, the selection of prediction intervals has a direct impact on model performance, and few works have systematically examined this factor under real-world conditions.

These three problems present key challenges to developing passenger flow forecasting models that are both accurate and generalizable in metro systems. Addressing them requires a spatial-temporal prediction framework that can (1) identify relevant training data patterns, (2) incorporate spatial correlations among stations, and (3) adapt flexibly to different prediction intervals. Our study aims to fill this gap by proposing a solution that jointly addresses all three aspects.

To address these challenges, we propose an enhanced deep learning model, termed the Spatial-Temporal Long Short-Term Memory (ST-LSTM) model, which leverages multi-source big data to learn spatial-temporal correlations of station passenger flows. Specifically, we design a temporal correlation learning module to mitigate the influence of weakly correlated historical data by identifying days with similar travel patterns and selectively incorporating them into the training process. To capture spatial correlations that are often ignored in conventional LSTM-based methods, we introduce a spatial correlation learning module that integrates origin-destination (OD) matrices, passenger flow data, and geographic information to quantify inter-station spatial relationships. Additionally, to examine the impact of prediction intervals, we evaluate the model under multiple time intervals (15, 30, and 60 minutes), demonstrating its adaptability across different operational scenarios. The proposed ST-LSTM model is validated on real-world datasets from the Nanjing and Chongqing metro systems and consistently outperforms baseline methods across all settings. In summary, the main contributions of this study are as follows:

- We propose a deep learning framework utilizing multi-source big data, based on an enhanced LSTM model, to predict short-term metro inbound and outbound passenger flows.
- We introduce a temporal correlation learning module to identify data with similar travel patterns, filtering out irrelevant historical data, effectively reducing the influence of irrelevant temporal inputs.
- We introduce a spatial correlation learning module that captures inter-station spatial correlations by combining OD matrices, flow statistics, and geographic information.
- We conduct extensive experiments under different prediction intervals to evaluate the model’s performance and demonstrate its practical applicability across time scales.

The remainder of this paper is organized as follows: In the “Related work” section, we review relevant literature pertaining to our research. Next, the “Preliminaries” section presents the problem statement and defines critical terms used throughout the study. In the “Methodologies” section, we detail the LSTM and ST-LSTM models. The “Experiment” section details the experimental setup and discusses results based on real-world data from the Nanjing and Chongqing Metro systems. Finally, the “Conclusion” section summarizes our findings, highlights contributions, and proposes future research directions.

## Related work

The task of short-term passenger flow prediction has been extensively researched. Initially, models relied heavily on classic linear prediction methods. Ahmed and Cook pioneered applying the autoregressive integrated moving average (ARIMA) model for short-term prediction of highway traffic flow [8]. Subsequently, regression time series models, such as various forms of the ARIMA model, have been widely employed. For instance, the multivariate ARIMA (ARIMAX) model demonstrated superiority in highway data prediction tasks [9]. Williams et al. introduced the seasonal autoregressive integrated moving average (SARIMA) model for modeling traffic data [10,11]. In the context of urban rail transit, Chen et al. applied the ARIMA model to predict passenger flow [12]. In addition to ARIMA, the Kalman filter model has gained popularity for its capability to handle data noise issues. Jiao et al. enhanced the traditional Kalman filter model by incorporating historical biases, Bayesian methods, and an error correction system to predict short-term rail transit passenger flows effectively [13].

However, traffic data exhibits randomness, time-varying dynamics, and high nonlinearity, posing challenges for traditional linear models in effectively handling such complexities and uncertainties. Consequently, there is a growing interest in advanced machine learning techniques, neural networks, and deep learning models like LSTM and convolutional neural networks for short-term passenger flow prediction.

Advanced machine learning methods have emerged to address the nonlinear characteristics of traffic volume and passenger flow data. Liu and Yao proposed a subway passenger flow prediction model using a modified least squares support vector machine (LSSVM) [14]. Sengupta et al. employed a generalized Bayesian recurrent neural network to predict traffic flow, providing a framework that quantifies prediction uncertainty [15]. Tianwei et al. introduced an enhanced K-nearest neighbor algorithm, optimizing the neighbor-matching mechanism for short-term passenger flow prediction, particularly beneficial for new subway stations [16].

Various neural network models have been developed for short-term passenger flow prediction. Traditional feedforward neural networks excel in capturing intricate nonlinear relationships without requiring prior domain knowledge [17]. To enhance the understanding of spatial-temporal characteristics in traffic data, Jing and Yin utilized a neural network model for predicting short-term inbound and outbound passenger flows based on multi-source railway big data [18]. In another study, Li et al. introduced a novel dynamic radial basis function (RBF) neural network designed to predict short-term outbound passenger flows. Their research demonstrated that this approach significantly enhanced the accuracy of short-term passenger flow predictions [19].

Modern deep learning models, characterized by complex artificial neural networks, have found extensive applications in natural language processing, speech recognition, and computer vision. Their capability to capture intricate nonlinear relationships from vast datasets has led to remarkable predictive performance advancements in transportation [20]. For instance, LSTM-based models have been utilized to forecast short-term passenger flows. Addressing data volatility concerns, Yang et al. developed a hybrid prediction model for subway inbound passenger flow using wavelet analysis and LSTM (Wave-LSTM) [6]. Similarly, Bharti et al. proposed a model combining particle swarm optimization (PSO) with a bidirectional LSTM (Bi-LSTM) neural network for short-term traffic flow prediction, effectively capturing data periodicity and volatility [21]. However, these methods sometimes fail to account for variations in historical passenger travel patterns, introducing noise in temporal correlation learning. To mitigate this issue, Hao et al. introduced an end-to-end deep learning framework focusing exclusively on relationships between station passenger flows within the same period (daily/weekly intervals) [1]. Nonetheless, this approach necessitates a substantial dataset. Moreover, these methods often do not explicitly incorporate spatial information. He et al. introduced a deep learning framework for predicting inbound and outbound passenger flows across different regions to address this gap. This framework employs regional segmentation and approximation to divide irregular regions into regular grids, utilizing convolution operations to capture spatial dependencies [22]. Similarly, Zhao et al. used convolutional neural networks and self-attention mechanisms to extract spatial features from traffic flow data for short-term traffic flow prediction [23]. Wang et al. developed a temporal graph attention convolutional neural network (TGACN) to extract spatial-temporal correlations in station passenger flow [24]. A notable challenge in these methods is the inclusion of redundant data from weakly correlated regions, which can diminish model performance during spatial correlation extraction. To address this, Liu et al. focused on spatial correlations between stations with short travel times when predicting subway passenger flows [2]. Similarly, Yang et al. advanced a model for predicting subway exit passenger flows based on an improved LSTM, concentrating on spatial-temporal correlations between stations on the same line [5]. However, these approaches may overlook relationships between attracting and diverting passengers at adjacent but poorly connected stations and hidden connections between geographically distant areas.

To further address the limitations of LSTM- and CNN-based models in capturing complex spatial structures, Graph Neural Networks (GNNs) have recently been introduced in the context of short-term passenger flow prediction. GNNs are particularly effective at modeling topological relationships among metro stations and learning high-level spatial dependencies. For example, Lu et al. proposed an Adaptive Multi-view Fusion Graph Neural Network (AMFGNN), which constructs and integrates various graph views (e.g., physical topology, line accessibility, spatial distance) to enhance spatial interaction modeling using attention mechanisms [25]. Wang et al. proposed KoopGCN, which combines Koopman theory and graph convolution to predict non-stationary traffic flow, achieving superior accuracy under varying conditions [26]. Zhang et al. proposed STG-GAN, a spatiotemporal graph adversarial network that improves prediction accuracy and efficiency by jointly modeling spatial and temporal constraints [27]. These studies demonstrate the modeling power of GNNs in complex spatial networks. However, most GNN-based models function as black boxes and offer limited interpretability, making it difficult to explicitly identify which stations contribute most to prediction results.

In addition to advancements in modeling spatial dependencies, recent studies have increasingly recognized that the choice of prediction time intervals can significantly affect model accuracy. For instance, Lu et al. proposed the Mul-DesLSTM model, which fuses data across multiple time intervals using a dense residual structure, leading to significantly improved prediction accuracy compared to single-interval models [28]. Building on this, Lu et al. also developed MST-GRT, a Multi-Spatio-Temporal Convolutional Neural Network that aggregates multi-interval data through multi-graph neural networks and dilated temporal convolutions to capture both temporal and spatial dependencies [29]. Zhang et al. further investigated this aspect by introducing a hybrid ResNet-GCN-LSTM model (ResLSTM), which was evaluated across 10-, 15-, and 30-minute intervals. Their findings showed that prediction accuracy improves with coarser time intervals [30]. Moreover, the IPF-HMGNN model integrates multi-resolution information by aligning disaggregated and aggregated flows within a hierarchical message-passing framework, thereby improving model robustness across time scales [31].

In summary, certain studies have overlooked the dynamic nature of passenger travel patterns over time, while others encounter challenges with redundant or incomplete spatial correlation information. In addition, selecting an appropriate temporal interval is crucial, as it directly affects the model’s ability to capture meaningful spatiotemporal patterns. With the continued growth in subway passenger demand and advancements in big data technologies, the application of deep learning methods in passenger flow prediction has become increasingly pertinent. Accordingly, there is a critical need to develop a dedicated passenger flow prediction model for urban rail transit that comprehensively incorporates both spatial and temporal characteristics of subway passenger travel data.

## Preliminaries

This study aims to predict the short-term inbound and outbound passenger flow for each metro station individually. Specifically, using historical AFC data and auxiliary information, our model estimates the number of passengers entering and exiting a given station during the upcoming 15-minute interval. An example dataset from the Nanjing Metro, illustrating the format for collecting passenger flow data, is presented in Table 1.

**Table 1:** Dataset of inbound and outbound passenger volumes at Nanjing Metro stations.

ID	Original time	Station code	Inbound flow	Outbound flow	Station name	Line ID
1	6:00:00	19	14	9	Olympic Stadium East Station	02
2	6:15:00	55	22	34	China Medicine University Station	03
...	...	...	...	...	...	...

We can utilize a one-dimensional time series to depict the inbound/outbound passenger flows at the station:

$$F_{s}^{d}=\{f_{s}^{\;\;d,1},f_{s}^{\;\;d,2},\ldots ,f_{s}^{\;\;d,t-i},\ldots ,f_{s}^{\;\;d,t-1},f_{s}^{\;\;d,t}\}$$ (1)

where $F_{s}^{d}$ represents the set of inbound/outbound passenger flows at station *s* across all time periods on the *d*-th day, and $f_{s}^{\;\;d,t}$ represents the inbound/outbound passenger flow at station *s* during the *t*-th time period on the *d*-th day.

Passengers’ travel patterns vary over time and across locations. Therefore, achieving highly accurate prediction results necessitates considering both the temporal dynamics of station passenger flows and the spatial correlations between stations. In URT systems, passenger flows between different stations mutually influence each other. For instance, geographically adjacent stations have relative relationships in attracting and redirecting passengers due to similarities in their surroundings and functional roles. Moreover, stations located in areas with similar functions, such as commercial or residential zones, may exhibit comparable travel patterns despite being geographically distant. Considering these factors, we construct a matrix that integrates the temporal and spatial characteristics of historical passenger flow data from various stations. The day is divided into *h* periods, as illustrated in Eq (2). Each row of the matrix represents the temporal characteristics of passenger flows at a station, while each column captures the spatial characteristics of passenger flows between stations.

$$F={\left[\begin{array}{cccc} F_{S_{1}}^{1} & F_{S_{1}}^{\;2} & \ldots & m_{S_{1}}^{d}\\ F_{S_{2}}^{1} & F_{S_{2}}^{2} & \ldots & F_{S_{2}}^{d}\\ \vdots & \vdots & \ddots & \vdots \\ F_{S_{n}}^{1} & F_{S_{n}}^{2} & \ldots & F_{S_{n}}^{d} \end{array}\right]}_{S_{n}\times d}\;={\left[\begin{array}{ccccccc} f_{S_{1}}^{\;\;1,1} & f_{S_{1}}^{\;\;1,2} & \ldots & f_{S_{1}}^{\;\;d-y,H-1} & f_{S_{1}}^{\;\;d-y,H} & \ldots & f_{S_{1}}^{\;\;d,t}\\ f_{S_{2}}^{\;\;1,1} & f_{S_{2}}^{\;\;1,2} & \ldots & f_{S_{2}}^{\;\;d-y,H-1} & f_{S_{2}}^{\;\;d-y,H} & \ldots & f_{S_{2}}^{\;\;d,t}\\ \vdots & \vdots & & & & & \vdots \\ f_{S_{n}}^{\;1,1} & f_{S_{n}}^{\;1,2} & \ldots & f_{S_{n}}^{\;\;d-y,H-1} & f_{S_{n}}^{\;\;d-y,H} & \ldots & f_{S_{n}}^{\;\;d,t} \end{array}\right]}_{S_{n}\times [h(d-1)+t]}$$ (2)

Moreover, using all historical passenger flow data from every station in the rail transit network to predict flows at a specific station is unnecessary and overly complex. For instance, in Nanjing Metro, travel patterns significantly differ between weekdays, weekends, and holidays, impacting passenger behavior. Additionally, stations on different lines, with diverse surroundings and distant geographic locations, often exhibit minimal flow correlations. Therefore, filtering historical data to match the day’s travel patterns and identifying stations strongly correlated with the station of interest is crucial. This motivates us to construct a more informative matrix, denoted as *F*.

To develop an effective prediction model that accommodates the spatial-temporal characteristics of passenger flows in urban rail transit stations while ensuring stability and flexibility, we propose an enhanced spatial-temporal LSTM model (ST-LSTM). This model predicts inbound and outbound passenger flows at stations using inputs such as inbound and outbound flows, OD matrices, geographic coordinates, and operational data from the rail transit network. The model outputs the predicted flows for the station. Table 2 summarizes the symbols used in our model.

**Table 2:** Symbols explanation in the model.

Notations	Definitions
Variables	
$f_{s}^{\;\;d,t}$	Inbound/outbound passenger flow of station *s* at time slot *t* on day *d*
$f_{i}^{u}$	Total inbound/outbound passenger ridership of station *i* on day *u*
*p*_*i*_	Trend correlation between station *i* and the station to be predicted (station *k*)
*m*_*ij*_	Total OD flow from station *i* to station *j* in the training set
*f*_*k*(*inflow*)_	Total inbound passenger flow of station *k* in the training set
*f*_*k*(*outflow*)_	Total outbound passenger flow of station *k* in the training set
*q*_*i*_	Passenger flow contribution of station *i* to the station *k*
$v_{i}$	Variable obtained after max-min normalization of *q*_*i*_
*f*_*i*_	Total passenger flow of station *i* in the training set
*r*_*i*_	Location correlation between station *i* and station *k*
*g*_*i*_	Variable obtained after max-min normalization of *r*_*i*_
*d*_*i*,*k*_	Geographical distance between station *i* and station *k*
*z*_*i*_	Ultimate measure of spatial correlation between station *i* and station *k*
Sets	
$F_{s}^{d}$	Set of inbound/outbound passenger flows entering/exiting station *s* in different slots on day *d*
$C_{b}^{d}$	Set of operating days belonging to class *b*
*F*_*k*_	Set of inbound/outbound passenger flow of station *k* in the training set
*L*	Set of all class *l* operating days before day *d*
$H_{S_{x}}^{L}$	Set of historical inbound/outbound passenger flow of station *S*_*x*_ on days in set *L*
$R_{S_{x}}^{d}$	Set of real-time inbound/outbound passenger flow of station *S*_*x*_ on day *d*
Matrices	
*Fdata*	Matrix of daily inbound/outbound passenger flows of all stations in the training set
*M*	Total OD matrix of the training set
Parameters	
*h*	Number of time slots that one day is divided into
*u*	Number of days for which the training set contains data
*n*	Number of stations in urban rail transit
*b*	Number of classifications obtained by clustering operational days
*x*	Number of stations that have a higher spatial correlation with station *k*
*a*	Number of days contained in set *L*

## Methodologies

### Long short-term memory (LSTM) networks

**Fig 1:**
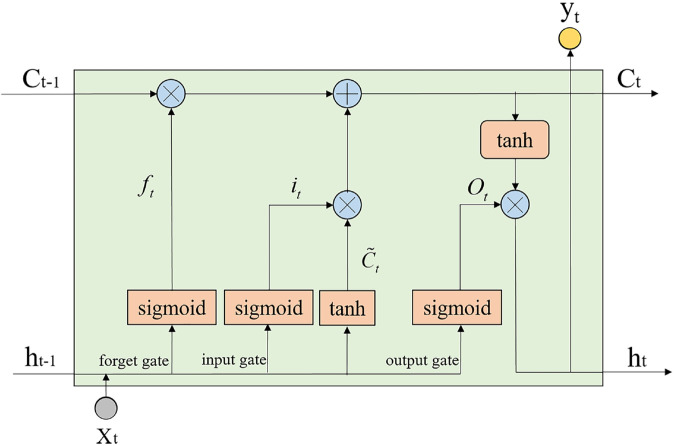
The basic structure of a LSTM unit.

Recurrent Neural Networks (RNNs) are designed to process sequential data by linking current outputs with previous inputs and outputs. However, traditional RNNs often face issues such as vanishing or exploding gradients, especially when dealing with long sequences. To overcome these challenges, Hochreiter and Schmidhuber introduced an enhanced RNN variant known as LSTM, which excels in capturing long-range dependencies [32]. The fundamental architecture of LSTM is illustrated in Fig 1. Unlike simple RNNs, LSTM integrates a mechanism for evaluating information relevance, comprising three gates: the forget gate, the input gate, and the output gate. Moreover, LSTM incorporates a cell state denoted as *C*, which facilitates long-term information retention. The operation of LSTM can be described in four main steps:

Step 1: Upon receiving new input *x*_*t*_, the LSTM’s forget gate assesses which existing information to discard. This process is defined by Eq (3):

$$f_{t(forgetgate)}=sigmoid(W_{fh}h_{t-1}+W_{fx}x_{t}+b_{f})$$ (3)

where *W*_*fh*_ and *W*_*fx*_ represent the weights of the forget gate, *b*_*f*_ is the bias of the forget gate, and *h*_*t*−1_ is the hidden state at time *t*–1. The *sigmoid* function is a commonly used nonlinear activation function, $sigmoid(x)=\frac{1}{1+e^{-x}}$.

Step 2: The input gate regulates the amount of new input information *x*_*t*_ that should be incorporated into the current cell state *C*_*t*_ to prevent the storage of irrelevant data. This process involves two main tasks: (*i*.) The sigmoid layer determines the amount of information to retain:

$$i_{t(inputgate)}=sigmoid(W_{ih}h_{t-1}+W_{ix}x_{t}+b_{i})$$ (4)

(*ii*.) The Tanh activation function generates the candidate set $\tilde{C_{t}}$ as follows:

$$\tilde{C_{t}}=tanh(W_{ch}h_{t-1}+W_{cx}x_{t}+b_{c})$$ (5)

where *W*_*ih*_, *W*_*ix*_ and *b*_*i*_ denote the weight and bias of the input gate, respectively. *W*_*ch*_, *W*_*cx*_ and *b*_*c*_ represent the weight and bias parameters of the candidate cell state. *Tanh* is another common nonlinear activation function, $tanh(x)=\frac{e^{x}-e^{-x}}{e^{x}+e^{-x}}$.

Step 3: The previous two steps are integrated to update the cell state *C*_*t*−1_ from the previous stage. The updated cell state *C*_*t*_ at this stage represents the process of discarding irrelevant information and incorporating new information, as illustrated by Eq (6):

$$C_{t}=f_{t}⊙C_{t-1}+i_{t}⊙\tilde{C_{t}}$$ (6)

where ⊙ represents the Hadamard product.

Step 4: The output gate determines the value of the current output *h*_*t*_, which is derived from the current cell state *C*_*t*_. This process is described by Eq (7) and Eq (8):

$$o_{t(outputgate)}=sigmoid(W_{oh}h_{t-1}+W_{ox}x_{t}+b_{o})$$ (7)

$$h_{t}=o_{t}⊙tanh(C_{t})$$ (8)

where *W*_*oh*_, *W*_*ox*_ and *b*_*o*_ denote the weight and bias term of the output gate, respectively.

### Model development

Typically, LSTM models utilize historical data from the target station as input. However, when predicting passenger flow, LSTM models often consider data from only one station, ignoring the interactions between stations. This method fails to accurately capture passenger flow patterns in complex rail transit networks, such as those in major cities like Nanjing, where flows between stations are closely interconnected. Consequently, LSTM models have inherent limitations. Our proposed model overcomes these limitations using a two-dimensional matrix incorporating historical passenger flow data across various times and stations. This approach effectively captures passenger flow data’s temporal and spatial characteristics.

**Fig 2:**
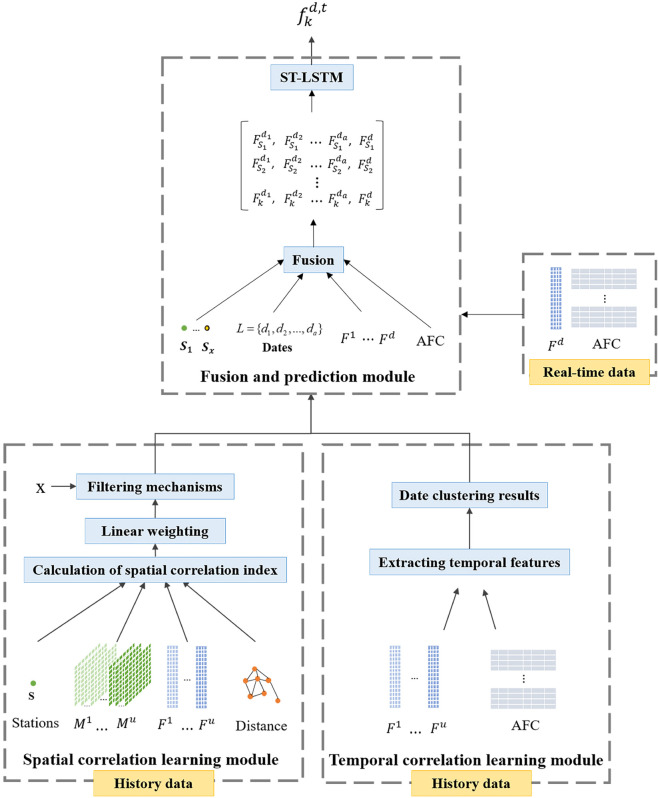
Overall framework of ST-LSTM framework.

Fig 2 shows the framework of our enhanced Long Short-Term Memory (ST-LSTM) prediction method. The framework includes three main components: the temporal correlation learning module, the spatial correlation learning module, and the fusion prediction module. The temporal Correlation Learning Module groups operating days based on historical passenger flow patterns. It selects historical data with similar patterns to the predicted day and defines the input data timeframe for the other modules. The spatial Correlation Learning Module uses three indicators to evaluate spatial correlations between stations, identifying those with strong connections to the target station. The fusion Prediction Module combines historical and real-time data into a two-dimensional matrix for prediction. The model outputs the target station’s predicted inbound/outbound passenger flow. The following subsections will provide a detailed explanation of these modules.

#### Temporal correlation learning module.

Previous studies often use continuous and extensive historical data to predict station passenger flow [6,21]. However, this approach can include weakly correlated information from days with different travel patterns. To solve this, we designed a time correlation learning module to filter more effective training data, improving computational efficiency and prediction accuracy.

To find training data with similar travel patterns, we classify operating days based on the temporal characteristics of passenger flow. We extract the entire network’s inbound/outbound passenger flow matrix from a training set with *u* days of data, using each operating day (i.e., 24 hours) as the interval. Assuming there are *n* stations in the URT network, this results in an n×u matrix, as shown in Eq (9).

$$FData={\left[\begin{array}{ccccc} f_{1}^{\;\;1} & f_{1}^{\;\;2} & f_{1}^{\;\;3} & \ldots & f_{1}^{\;\;u}\\ f_{2}^{\;\;1} & f_{2}^{\;\;2} & f_{2}^{\;\;3} & \ldots & f_{2}^{\;\;u}\\ \vdots & \vdots & \vdots & \ddots & \vdots \\ f_{i}^{\;\;1} & f_{i}^{\;\;2} & f_{i}^{\;\;3} & \ldots & f_{i}^{\;\;u}\\ \vdots & \vdots & \vdots & \ddots & \vdots \\ f_{n}^{\;\;1} & f_{n}^{\;\;2} & f_{n}^{\;\;3} & \ldots & f_{n}^{\;\;u} \end{array}\right]}_{n\times u}$$ (9)

where $f_{i}^{u}$ represents the daily inbound/outbound passenger flow of station *i* on day *u*.

Next, we apply an improved K-means clustering algorithm based on the elbow method [33] to cluster the matrix *FData*. This method determines the optimal number of clusters, *b*, and divides the operating days into *b* categories, represented by *C*:

$$C=\{C_{1},C_{2},\ldots ,C_{b}\}$$ (10)

where *C*_*b*_ represents the set of operating days belonging to category *b*.

#### Spatial correlation learning module.

Previous studies have explored spatial correlations in station passenger flow data using various methods. Some use convolutional neural networks to capture spatial effects across the network [22,24], while others focus on neighboring areas [2,5]. Unlike these approaches, we introduce a comprehensive indicator to evaluate spatial correlation between stations across the entire network, considering both passenger travel patterns and station geographic locations.

Using inbound/outbound passenger flow data, OD matrix, operational data, and geographic data, we introduce three indicators, *p*, *q*, and *r*, to measure station spatial correlation. Assume the target station is *k*. First, *p*_*i*_ represents the trend correlation between station *i* and station *k*, derived from the Pearson coefficient:

$$p_{i}=|\frac{Cov(F_{k},F_{i})}{\sigma (F_{k})\sigma (F_{i})}|$$ (11)

where *F*_*k*_ represents the set of inbound/outbound passenger flow for station *k* in the training set, *Cov* is the covariance function, and *σ* is the standard deviation function. The range of *p*_*i*_ is [0,1]; a higher *p*_*i*_ indicates a stronger trend correlation between the stations’ passenger flows.

Assuming there are *n* stations in the URT network, N={1,2,…,i,…,n}. The OD matrix *M* is expressed in Eq (12):

$$M={\left[\begin{array}{ccccccc} 0 & m_{1,2} & \ldots & m_{1,j} & \ldots & m_{1,n-1} & m_{1,n}\\ m_{2,1} & 0 & \ldots & m_{2,j} & \ldots & m_{2,n-1} & m_{2,n}\\ \vdots & \vdots & \ddots & \vdots & \ddots & \vdots & \vdots \\ m_{j,1} & m_{j,2} & \ldots & 0 & \ldots & m_{j,n-1} & m_{j,n}\\ \vdots & \vdots & \ddots & \vdots & \ddots & \vdots & \vdots \\ m_{n-1,1} & m_{n-1,2} & \ldots & m_{n-1,j} & \ldots & 0 & m_{n-1,n}\\ m_{n,1} & m_{n,2} & \ldots & m_{n,j} & \ldots & m_{n,n-1} & 0 \end{array}\right]}_{n\times n}$$ (12)

where *m*_*i*,*j*_ represents the total OD flow from station *i* to station *j* in the training set.

If *f*_*k*(*inbound*)_ is the total inbound flow and *f*_*k*(*outbound*)_ is the total outbound flow for station *k* in the training set, they are expressed in Eq (13):

$$f_{k(inbound)}=\sum_{j=1}^{n}m_{k,j}$$ (13a)

$$f_{k(outbound)}=\sum_{i=1}^{n}m_{i,k}$$ (13b)

Here, *q*_*i*_ represents the passenger flow contribution of station *i* to station *k*:

$$q_{i(inbound)}=\frac{m_{k,i}}{f_{k(inbound)}}+\frac{m_{k,i}}{f_{i(outbound)}}$$ (14a)

$$q_{i(outbound)}=\frac{m_{i,k}}{f_{i(inbound)}}+\frac{m_{i,k}}{f_{k(outbound)}}$$ (14b)

where *q*_*i*(*inbound*)_ is used for inbound prediction and *q*_*i*(*outbound*)_ for outbound prediction. The maximum value is *q*_*max*_, and the minimum is *q*_*min*_.

Spatial correlation decreases with distance [2]. Inspired by the distance decay theory [34,35], indicator *r* represents the interaction intensity between stations, as shown in Eq (15):

$$r_{i}=\frac{f_{i}f_{k}}{d_{i,k}^{2}},f_{i}=f_{i(inbound)}+f_{i(outbound)};f_{k}=f_{k(inbound)}+f_{k(outbound)}$$ (15)

where *r*_*i*_ is the interaction strength between station *i* and station *k*, *f*_*i*_ is the total passenger flow of station *i* in the training set, and *d*_*i*,*k*_ is the geographical distance between stations *i* and *k*.

The maximum value of *r* is *r*_*max*_, and the minimum is *r*_*min*_. Indicators *p*, *q*, and *r* are normalized to ensure they are on the same scale, as shown in Eq (16):

$$v_{i}=\frac{q_{max}-q_{i}}{q_{max}-q_{min}}$$ (16a)

$$g_{i}=\frac{r_{max}-r_{i}}{r_{max}-r_{min}}$$ (16b)

where *q*_*max*_, *q*_*min*_, *r*_*max*_, and *r*_*min*_ represent the maximum and minimum values of the indicators, and *v*_*i*_ and *g*_*i*_ are the normalized values. Then, the three indicators are combined into a single indicator using the linear weighted compromise method. The final indicator is shown in Eq (17):

$$z_{i}=\omega_{1}\cdot p_{i}+\omega_{2}\cdot v_{i}+\omega_{3}\cdot g_{i}$$ (17)

After sorting the *z* values in descending order, the top *x* stations with the strongest spatial correlation to the target station are identified as *S*_1_, *S*_2_, ..., *S*_*x*_.

#### Fusion and prediction module.

After extracting the spatial-temporal characteristics of station passenger flow from the previous modules, the data must be fused before making predictions. First, the historical data is processed. The time correlation learning module divides the days into *b* categories, while the spatial correlation learning module identifies the top *x* stations with the highest spatial correlation. Suppose the day to be predicted (denoted as *d*) belongs to category *l*, i.e., $d\in C_{l}$ (where *C*_*l*_ is the set of operating days in category *l*). For clarity, let $L=d_{1},d_{2},\ldots ,d_{a}$ represent all days in category *l* before day *d*, with subscripts indicating their sequence. The historical data to be used is given by Eq (18):

$$H_{S_{1}}^{L}=\{F_{S_{1}}^{\;d_{1}},F_{S_{1}}^{\;d_{2}},\ldots ,F_{S_{1}}^{\;d_{a}}\}$$ (18a)

$$H_{S_{2}}^{L}=\{F_{S_{2}}^{\;d_{1}},F_{S_{2}}^{\;d_{2}},\ldots ,F_{S_{2}}^{\;d_{a}}\}$$ (18b)

$$H_{S_{x}}^{L}=\{F_{S_{x}}^{\;d_{1}},F_{S_{x}}^{\;d_{2}},\ldots ,F_{S_{x}}^{\;d_{a}}\}$$ (18c)

$$H_{k}^{L}=\{F_{k}^{\;d_{1}},F_{k}^{\;d_{2}},\ldots ,F_{k}^{\;d_{a}}\}$$ (18d)

where $H_{S_{x}}^{L}$ represents the historical inbound/outbound passenger flow of station *S*_*x*_ on days in set *L*, containing *a* elements. $F_{S_{x}}^{d_{a}}$ is the inbound/outbound flow of station *S*_*x*_ on day *d*_*a*_, with *h* elements.

Next, real-time data is processed using the spatial correlation module’s output. Assuming the inbound/outbound passenger flow of station *k* in time period *t* on day *d* is to be predicted, the real-time passenger flow data is expressed as:

$$R_{S_{1}}^{d}=\{f_{S_{1}}^{\;\;d,1},f_{S_{1}}^{\;\;d,2},\ldots ,f_{S_{1}}^{\;\;d,t-1}\}$$ (19a)

$$R_{S_{2}}^{d}=\{f_{S_{2}}^{\;\;d,1},f_{S_{2}}^{\;\;d,2},\ldots ,f_{S_{2}}^{\;\;d,t-1}\}$$ (19b)

$$R_{S_{x}}^{d}=\{f_{S_{x}}^{\;\;d,1},f_{S_{x}}^{\;\;d,2},\ldots ,f_{S_{x}}^{\;\;d,t-1}\}$$ (19c)

$$R_{k}^{d}=\{f_{k}^{\;\;d,1},f_{k}^{\;\;d,2},\ldots ,f_{k}^{\;\;d,t-1}\}$$ (19d)

Finally, the historical data is combined with the real-time data to form a two-dimensional matrix, as shown in Eq (20). This matrix is input into the prediction module, resulting in the predicted inbound/outbound passenger flow for station *k*.

$$Input={\left[\begin{array}{cc} H_{S_{1}}^{L} & R_{S_{1}}^{d}\\ H_{S_{2}}^{L} & R_{S_{2}}^{d}\\ \vdots & \vdots \\ H_{S_{x}}^{L} & R_{S_{x}}^{d}\\ H_{k}^{L} & R_{k}^{d} \end{array}\right]}_{\left(x+1)\times (ah+t-1\right)}$$ (20)

#### Optimization and training.

The model’s output is the predicted inbound/outbound passenger flow. During training, the goal is to minimize the error between the predicted and actual passenger flow. We use the mean square error (MSE) as the loss function, defined as follows:

$$L(\theta )=\frac{1}{n}\sum_{k=1}^{n}(f_{k}^{\;\;d,t}-{\hat{f}}_{k}^{\;\;d,t}{)}^{2}$$ (21)

where ${\hat{f}}_{k}^{\;\;d,t}$ s the predicted inbound/outbound passenger flow, and $f_{k}^{\;\;d,t}$ is the actual inbound/outbound passenger flow. The symbol *θ* represents all learnable parameters in the network, which are optimized using the back-propagation algorithm and the Adam optimizer [36].

## Experiment

In this section, we perform extensive experiments on two real-world datasets to evaluate and compare the proposed method with multiple baseline methods. We analyze the results from various perspectives.

### Data description

We used two datasets: Nanjing Metro and Chongqing Metro, as shown in Table 3. The Nanjing Metro dataset (MetroNJ2018) contains over 50 million passenger card swipe records from March 2018, covering 175 stations. The Chongqing Metro dataset (MetroCQ2019) includes over 70 million card swipe records from May 2019, covering 177 stations. Each record includes the card number, entry and exit station names, as well as swipe time. Passenger flow data is extracted at 15-minute intervals, with statistics collected from 5:00 to 23:00, resulting in 72 timestamps per day.

**Table 3:** Data description.

Description	MetroNJ2018	MetroCQ2019
Data	March 1, 2018 to March 31, 2018	May 1, 2019 to May 31, 2019
Data record	52 million	71 million
Station number	175	177
Time interval	15min	15min
Time	05:00 to 23:00	05:00 to 23:00
Timestamp number	72	72

Due to computational resource limitations, evaluating the model on all stations in the dataset is impractical. Therefore, we use stratified sampling to select station samples for model verification. Stations are categorized into three levels based on average daily passenger flow: large, medium, and small. We randomly select 20% of stations from each level for analysis. The last seven days of data are used for testing, while the remaining data is used for training.

### Experiment settings

To accelerate model learning and convergence, all passenger flow data were standardized using the min-max method and scaled to the range [0,1], as shown in Eq (22).

$$s=\frac{f-min(f)}{max(f)-min(f)}$$ (22)

After parameter tuning, our model uses two hidden layers with 32 units each. The Adam optimizer was applied with a learning rate 0.0005 and a batch size 12. We also tuned parameters for the comparison methods. Our model was implemented using PyTorch 1.1 in Python and ran on an NVIDIA GeForce RTX 3060 Laptop GPU.

In this study, we used two evaluation metrics to assess our model and the baselines: root mean square error (RMSE) and mean absolute error (MAE).

$$RMSE=[\frac{1}{\varepsilon }\sum_{i=1}^{\varepsilon }(|f_{i}-{\hat{f}}_{i}|{)}^{2}{]}^{\frac{1}{2}}$$ (23)

$$MAE=\frac{1}{\varepsilon }\sum_{i=1}^{\varepsilon }|f_{i}-{\hat{f}}_{i}|$$ (24)

where *ε* is the total number of predicted values, *f*_*i*_ is the actual inbound/outbound passenger flow, and ${\hat{f}}_{i}$ is the predicted inbound/outbound passenger flow.

### Methods for comparison

We compared the proposed ST-LSTM model with seven classical traffic prediction models to evaluate our model’s performance. Additionally, we built two other models based on ST-LSTM to demonstrate the effectiveness of the temporal and spatial correlation learning modules. Except for the control component, all other parameters are the same as in ST-LSTM. The comparison methods are detailed below:

- Long short-term memory (LSTM) [37]: A recurrent neural network for sequence data. It has two hidden layers with 32 units each, a learning rate 0.0005, and a batch size 12.
- Bidirectional LSTM (Bi-LSTM) [38]: Adds a bidirectional layer to LSTM. Both layers have two hidden layers with 32 units each, a learning rate of 0.0005, and a batch size of 12.
- Autoregressive Integrated Moving Average model (ARIMA) [39]: A classic time series model. ARIMA (2,1,0) was chosen for optimal prediction performance.
- Support Vector Regression (SVR) [40]: An application of support vector machine (SVM). Here, we used SVR with an RBF kernel for the experiment.
- Convolutional LSTM (ConvLSTM) [41]: Analyzes spatial-temporal data by replacing LSTM’s fully connected layer with a convolutional structure. It has two layers with 8 and 1 filters, respectively, a kernel size of 3 × 3, a learning rate of 0.0005, and a batch size of 12.
- Nonlinear AutoRegressive (NAR) [42]: Captures complex patterns in time series data using a neural network with two hidden layers of 32 neurons each and a delayed order 10.
- ST-LSTM (No temporal correlation): Removes the temporal correlation module to assess its impact. The input is continuous historical data.
- ST-LSTM (No spatial correlation): Removes the spatial correlation module to verify its effectiveness. The input is the station’s passenger flow data.
- ST-LSTM (ours): This is our whole model, as proposed in the “Methodologies” section. The model has two hidden layers of 32 units each, uses the Adam optimizer with a learning rate 0.0005 and a batch size 12.

### Experimental results

#### Overall performance.

**Fig 3:**
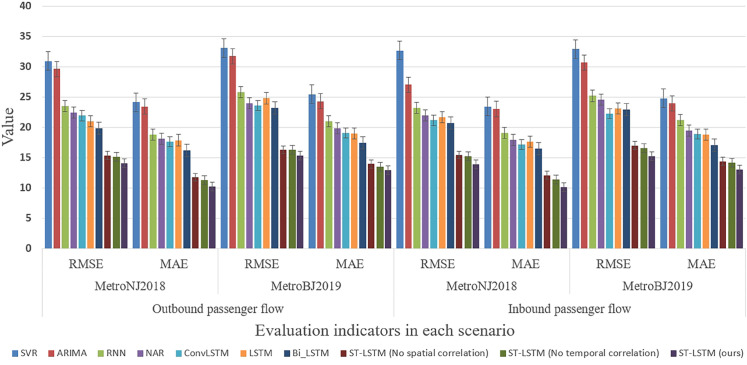
Comparison of different prediction models.

The experimental results are shown in Table 4 and Fig 3. The evaluation index represents the average prediction results for all stations, with the best result for each index highlighted in bold.

**Table 4:** Comparison of performances of different prediction models.

Models	Outbound passenger flow				Inbound passenger flow			
	MetroNJ2018		MetroCQ2019		MetroNJ2018		MetroCQ2019	
	RMSE	MAE	RMSE	MAE	RMSE	MAE	RMSE	MAE
SVR	30.941	24.163	33.118	25.459	32.674	23.442	32.937	24.803
ARIMA	29.655	23.445	31.751	24.301	27.068	23.037	30.722	23.957
RNN	23.549	18.836	25.847	21.036	23.201	19.113	25.207	21.218
NAR	22.451	18.104	24.033	19.855	22.010	17.899	24.573	19.433
ConvLSTM	21.936	17.670	23.591	19.104	21.164	17.192	22.306	18.868
LSTM	21.012	17.885	24.822	18.974	21.657	17.631	23.171	18.807
Bi-LSTM	19.849*	16.207*	23.254*	17.428*	20.703*	16.514*	22.904*	17.104*
ST-LSTM(No spatial correlation)	15.372	11.793	16.301	13.986	15.433	12.096	16.998	14.426
ST-LSTM(No temporal correlation)	15.164	11.336	16.351	13.498	15.215	11.376	16.631	14.207
ST-LSTM(ours)	**14.082**	**10.206**	**15.387**	**12.972**	**13.919**	**10.115**	**15.253**	**13.002**
Improvements	29.05%	37.03%	33.83%	25.57%	32.77%	38.75%	33.40%	23.98%

As seen in Table 4 and Fig 3, non-deep learning methods (such as SVR and ARIMA) perform poorly on the evaluation metrics compared to deep learning methods. This is likely due to their limited ability to capture the complex spatial-temporal characteristics necessary for predicting station passenger flow. Additionally, Bi-LSTM performs better than standard LSTM, as it considers the complete input sequence for predictions. However, the more complex ConvLSTM model performs worse than the standard LSTM, suggesting that more complex models are not necessarily better for short-term passenger flow prediction.

Our ST-LSTM model outperforms all compared models. Specifically, for outbound passenger flow prediction, our model surpasses other classic models by at least 29.05%, 37.03%, 33.83%, and 25.57% in RMSE and MAE on the MetroNJ2018 and MetroCQ2019 datasets, respectively. Inbound passenger flow prediction exceeds other models by at least 32.77%, 38.75%, 33.40%, and 23.98% in RMSE and MAE on the same datasets. These results validate the effectiveness of our ST-LSTM model.

The results also demonstrate the effectiveness of the temporal and spatial correlation learning modules. Compared with ST-LSTM (No spatial correlation), ST-LSTM improves RMSE by 8.39% and 5.61% on the MetroNJ2018 and MetroCQ2019 datasets for outbound flow prediction and by 9.81% and 9.87% for inbound flow prediction. Similarly, compared with ST-LSTM (No temporal correlation), ST-LSTM enhances RMSE by 7.14%, 5.90%, 8.52%, and 8.29% on the two datasets for both inbound and outbound flow predictions. The strong performance of the ST-LSTM model in various scenarios is attributed to its architecture, which integrates temporal and spatial correlation learning.

#### Prediction performances of individual stations.

**Fig 4:**
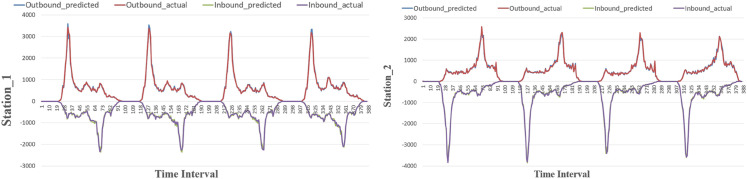
The results of selected stations in MetroNJ2018.

**Fig 5:**
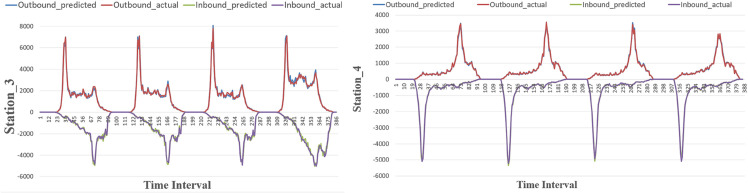
The results of selected stations in MetroCQ2019.

We selected several stations to evaluate the model’s performance in predicting individual station’s inbound and outbound passenger flow. The predicted passenger flow was compared with the actual values, as shown in Figs 4 and 5. Station_1 and Station_3 are typical commercial district stations, showing morning peaks in outbound flow and evening peaks in inbound flow. In contrast, Station_2 and Station_4 are typical residential district stations, with evening peaks in outbound flow and morning peaks in inbound flow. On both the MetroNJ2018 and MetroCQ2019 datasets, ST-LSTM accurately captures trend changes throughout the day for both inbound and outbound flows and even predicts peak flow accurately. In conclusion, our proposed model performs well in predicting passenger flow at the single station level on these two real datasets.

#### Performance under different temporal scenarios.

Through previous experiments, we demonstrated that ST-LSTM outperforms other models in various spatial scenarios. The experimental results for both inbound and outbound flows are similar, so we use outbound passenger flow prediction as an example to evaluate our model in different time scenarios.

First, we discuss the choice of prediction time interval. Various short-term passenger flow prediction studies use intervals of 5 to 60 minutes [1,2,27]. To compare with existing research, we tested ST-LSTM on MetroNJ2018 using different time intervals. The experimental results are shown in Table 5, with the best results for each evaluation index highlighted in bold.

**Table 5:** Performance comparison of ST-LSTM under different time intervals on MetroNJ2018.

Time interval	RMSE	MAE	Time requires
5min	19.133	13.738	268.013
10min	14.767	10.457	156.015
15min	**14.082**	**10.206**	127.391
30min	26.523	19.928	72.335
60min	44.963	33.783	26.21

Table 5 shows that larger time intervals require less computing time, and the model’s prediction accuracy is best with a 15-minute interval. A smaller interval may cause excessive fluctuations in passenger flow data, making it difficult for the model to learn patterns effectively. Conversely, the accuracy is poor with a 60-minute interval due to reduced training data, limiting the model’s learning ability.

Moreover, both prediction accuracy and computational efficiency should be considered when selecting the time interval. The experimental results indicate longer computation times with 5-minute and 10-minute intervals. To ensure efficiency in subsequent comparative experiments, we set the time intervals to 15, 30, and 60 minutes and tested the model’s prediction results accordingly. Tables 6 and 7 show the experimental results, with the best results for each indicator highlighted in bold.

**Table 6:** The prediction models’ performance under different time intervals on MetroNJ2018.

Models	15min		30min		60min	
	RMSE	MAE	RMSE	MAE	RMSE	MAE
SVR	30.941	24.163	56.143	43.901	69.027	53.976
ARIMA	29.655	23.445	55.946	43.687	67.992	53.094
RNN	23.549	18.836	43.424	34.733	59.373	50.490
NAR	22.451	18.104	39.451	31.983	58.017	49.562
ConvLSTM	21.936	17.670	36.246	30.046	56.941	47.957
LSTM	21.012	17.885	35.249	28.771	56.895	46.439
Bi-LSTM	19.849	16.207	33.324	26.516	52.364	43.826
ST-LSTM (No spatial correlation)	15.372	11.793	27.031	20.232	45.136	33.784
ST-LSTM (No temporal correlation)	15.164	11.336	27.026	20.168	45.122	33.505
ST-LSTM (ours)	**14.082**	**10.206**	**26.523**	**19.928**	**44.963**	**33.783**

**Table 7:** The prediction models’ performance under different time intervals on MetroCQ2019.

Models	15min		30min		60min	
	RMSE	MAE	RMSE	MAE	RMSE	MAE
SVR	33.118	25.459	58.265	45.886	71.149	55.961
ARIMA	31.751	24.301	58.135	45.645	70.181	55.052
RNN	25.847	21.036	45.557	36.596	63.506	51.353
NAR	24.033	19.855	41.896	33.909	61.108	51.077
ConvLSTM	23.591	19.104	39.846	32.739	59.752	50.456
LSTM	24.822	18.974	37.402	30.736	59.048	48.404
Bi-LSTM	23.254	17.428	35.958	28.095	55.497	44.017
ST-LSTM (No spatial correlation)	16.301	13.986	28.130	22.297	46.235	35.914
ST-LSTM (No temporal correlation)	16.351	13.498	28.049	22.125	46.145	35.901
ST-LSTM (ours)	**15.387**	**12.972**	**27.586**	**21.886**	**46.026**	**35.741**

Based on these experiments, we observe that among all evaluated prediction models, ST-LSTM consistently performs the best at each time interval. In contrast, non-deep learning models such as SVR and ARIMA perform the worst. ConvLSTM, LSTM, and Bi-LSTM show similar performance on both datasets, with Bi-LSTM standing out slightly. Additionally, all prediction models exhibit a similar trend: the highest prediction accuracy occurs at a 15-minute interval, while the worst performance is seen at a 60-minute interval. This result may be due to the reduced data available in 60-minute intervals, limiting the model’s learning capacity. These results emphasize the importance of choosing the right time interval and having sufficient data to capture complex travel patterns.

**Fig 6:**
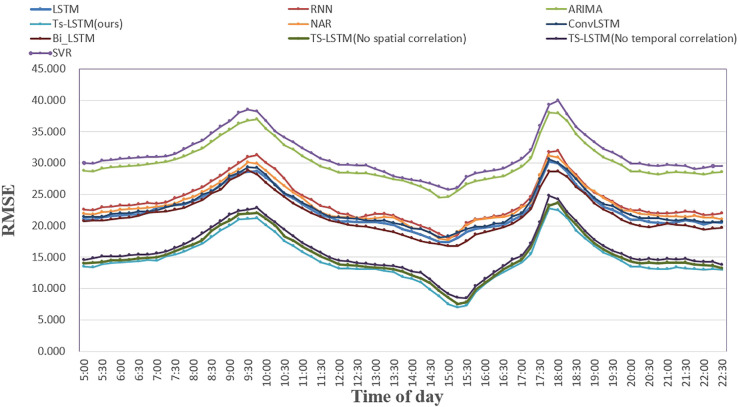
The performance of models in different time intervals.

To further evaluate our model’s performance across different times of the day, we divided the datasets into rush and non-rush hours. Weekday rush hours are defined as 7:00-9:00 and 17:00-19:00, with all other times being non-rush hours. Table 8 presents the experimental results, with the best results for each evaluation metric highlighted in bold. Fig 6 shows the RMSE trend for each prediction model on the MetroNJ2018 dataset over time. Key findings include:

- Despite increased travel demand and traffic unpredictability during peak hours, the ST-LSTM model consistently outperforms others, demonstrating its reliability and stability.
- Fig 6 shows that all models perform better during non-rush hours than rush hours. ST-LSTM maintains higher prediction accuracy than other models, showing strong adaptability across different scenarios.
- During non-rush hours, prediction performance is relatively stable for all models. However, there’s a noticeable performance gap during rush hours, with ST-LSTM better-capturing changes in passenger travel patterns. This highlights ST-LSTM’s stability and accuracy in predicting and adapting to high-demand periods.

**Table 8:** Performance comparison of prediction models under different temporal scenarios.

Models	MetroNJ2018				MetroCQ2019			
	Non-rush hours		Rush hours		Non-rush hours		Rush hours	
	RMSE	MAE	RMSE	MAE	RMSE	MAE	RMSE	MAE
SVR	28.314	22.682	34.016	26.863	32.436	24.667	36.538	28.848
ARIMA	27.181	21.522	32.128	25.157	30.370	24.480	34.317	27.115
RNN	22.792	17.247	25.735	21.067	24.925	20.210	28.868	24.030
NAR	21.066	17.396	24.386	20.456	22.562	18.456	27.865	23.446
ConvLSTM	20.850	16.009	23.080	19.645	23.852	17.295	27.065	22.846
LSTM	20.463	15.893	22.373	19.398	23.613	16.958	27.523	22.363
Bi-LSTM	18.697	15.190	21.456	18.546	22.196	16.085	25.499	21.456
ST-LSTM (No spatial correlation)	14.756	10.487	17.047	14.079	15.645	12.946	19.956	16.452
ST-LSTM (No temporal correlation)	14.298	10.290	16.456	13.785	15.300	12.846	19.685	16.263
ST-LSTM (ours)	**13.156**	**8.337**	**15.684**	**13.235**	**14.919**	**10.895**	**17.947**	**15.196**

#### Model interpretability.

To address the LSTM model’s limitation of capturing spatial relationships, we introduced a spatial correlation learning module to extract inter-station spatial correlations across the entire network. To assess the module’s impact and significance, along with the influence of varying *x* values (indicating the number of stations with higher spatial correlation with the station to be predicted) on model prediction performance, we focused on analyzing the Liuzhou East Road (LER) station within the MetroNJ2018 dataset. Table 9 summarizes the results obtained from Eqs (11)–((17).

**Table 9:** The top 10 stations with the highest *z*_*i*_ value.

Rank	Station Name	$p_{i}$	$v_{i}$	$g_{i}$	$z_{i}$
1	Wutang Square	0.989	0.289	0.706	1.843
2	Tianrun City	0.956	0.169	0.845	1.801
3	Shangyuan Gate	0.995	0.249	0.645	1.760
4	Nanjing Forestry University	0.947	0.189	0.747	1.733
5	Xinglong Street	0.988	0.210	0.608	1.684
6	Nanjing Station	0.912	0.289	0.480	1.586
7	Xuanwu Gate	0.965	0.130	0.598	1.574
8	Bell Tower	0.942	0.205	0.498	1.546
9	Shanghai Road	0.974	0.199	0.418	1.508
10	Xian Gate	0.986	0.198	0.389	1.496

In this table, *p*_*i*_ denotes the trend influence of station *i* on LER, $v_{i}$ represents the normalized passenger flow contribution from station *i*, and *g*_*i*_ reflects the normalized interaction intensity. Each station’s *p*_*i*_, $v_{i}$, and *g*_*i*_ are weighted linearly according to Eq (17). Notably, $\omega_{1}=\omega_{2}=1$ and $\omega_{3}=0.8$ were determined through parameter tuning to derive the spatial correlation index *z*_*i*_. The top 10 stations exhibiting the strongest correlation with LER are comprehensively ranked and presented in Table 9.

We conducted experiments varying the parameter *x* from 0 to 10 to assess its impact on model performance. Specifically, when *x* = 0, it is equivalent to removing the spatial correlation learning module from the model, effectively using ST-LSTM (No spatial correlation) for prediction. Results in Table 10 demonstrate that as *x* increases, the prediction accuracy of ST-LSTM also improves, validating the efficacy of the spatial correlation learning module. However, higher values of *x* correspond to increased computation time. Therefore, a balance between model performance and computational efficiency is crucial. We found that when the value of *x* is greater than 3, the improvement in model prediction performance significantly diminishes. Thus, we recommend setting the value of *x* to 3 for optimal model prediction.

**Table 10:** Performance of the model under different input conditions.

Input	Evaluation Indices		Improvements		Time requires(s)
x	RMSE	MAE	RMSE(%)	MAE(%)	
0	15.372	11.793	0.00	0.00	17.383
1	14.805	11.282	3.69	4.33	53.821
2	14.381	10.711	6.45	9.17	91.041
3	14.082	10.206	8.39	13.46	127.391
4	14.062	10.183	8.52	13.65	164.701
5	14.042	10.162	8.65	13.83	200.903
6	14.023	10.142	8.78	14.00	238.903
7	13.984	10.124	9.03	14.15	274.756
8	13.965	10.106	9.15	14.31	309.495
9	13.947	10.089	9.27	14.45	341.892
10	13.931	10.072	9.37	14.59	379.048

## Conclusion

This study establishes an improved spatial-temporal long short-term memory model (ST-LSTM) to predict short-term passenger flow at urban rail transit stations. The spatial and temporal characteristics of passenger flow were fully considered during the modeling process. The input of ST-LSTM includes the historical inbound and outbound passenger volume of each station, OD matrix, geographical distance data, and some operational data, with the output being the predicted inbound/outbound passenger volume of the target station. Using two real datasets, the Chongqing Subway and the Nanjing Subway, we evaluate the model’s performance in various spatial-temporal scenarios. Evaluations consistently show ST-LSTM outperforming baseline models, underscoring its robust capability to effectively extract and utilize spatial-temporal correlations in passenger flow data.

Key findings include: (1) Training models with data from days exhibiting similar travel patterns enhances computational efficiency and prediction accuracy; (2) To address LSTM’s spatial limitation in handling spatial information, we propose a spatial correlation learning module. This module leverages multi-source data to pre-select highly relevant stations across the network and integrate them into the model input. This innovative approach significantly improves forecast accuracy and has good interpretability; (3) The choice of prediction time interval significantly impacts model performance. A smaller time interval leads to more random station passenger flow data, reducing calculation efficiency. However, a larger time interval results in a reduced amount of available training data, potentially lowering model accuracy. Therefore, balancing computational efficiency and prediction accuracy and selecting an appropriate time granularity is crucial.

However, this study has certain limitations. First, since only one month of data is available, our study focuses on extracting the daily time correlation of passenger flow. As more extensive data becomes available, future studies could examine differences in traffic flow during different periods of the day to further improve forecast accuracy. Second, due to computational constraints, we adopt stratified sampling to evaluate model performance across the station network, which introduces some limitations in practical scalability.

Additionally, the model does not incorporate the effects of external factors such as weather conditions, holidays, or unexpected events due to the lack of annotated information in the current dataset. Although our temporal correlation learning module helps reduce the impact of weakly correlated historical data by selecting training samples with similar travel patterns, future research should include explicitly labeled special days or anomalous events to improve robustness and adaptability.

Moreover, this study is based on pre-pandemic data (2018–2019). Since travel behaviors have shifted considerably in the post-COVID era, future research will incorporate more recent datasets to compare station-level usage patterns and evaluate the model’s adaptability to emerging mobility dynamics.
